# Secretion and Uptake of α-Synuclein Via Extracellular Vesicles in Cultured Cells

**DOI:** 10.1007/s10571-018-0622-5

**Published:** 2018-10-04

**Authors:** Gabriel Gustafsson, Camilla Lööv, Emma Persson, Diana F. Lázaro, Shuko Takeda, Joakim Bergström, Anna Erlandsson, Dag Sehlin, Leonora Balaj, Bence György, Martin Hallbeck, Tiago F. Outeiro, Xandra O. Breakefield, Bradley T. Hyman, Martin Ingelsson

**Affiliations:** 1 Department of Public Health and Caring Sciences/Geriatrics, Uppsala University, Uppsala, Sweden; 2 Department of Neurology, Massachusetts General Hospital, Charlestown, USA; 3 Department of Radiology, Massachusetts General Hospital, Charlestown, USA; 4 Neuroscience Program, Harvard Medical School, Boston, USA; 5 Department of Pathology, Department of Clinical and Experimental Medicine, Linköping University, Linköping, Sweden; 6 Department of Experimental Neurodegeneration, University Medical Center Göttingen, Göttingen, Germany; 7 Max Planck Institute for Experimental Medicine, Göttingen, Germany; 8 Institute of Neuroscience, The Medical School, Newcastle University, Framlington Place, Newcastle Upon Tyne, NE2 4HH UK

**Keywords:** Alpha-synuclein, Parkinson’s disease, Alpha-synuclein oligomers, Human neuroblastoma, Extracellular vesicles, Exosomes

## Abstract

In Parkinson’s disease and other Lewy body disorders, the propagation of pathology has been accredited to the spreading of extracellular α-synuclein (α-syn). Although the pathogenic mechanisms are not fully understood, cell-to-cell transfer of α-syn via exosomes and other extracellular vesicles (EVs) has been reported. Here, we investigated whether altered molecular properties of α-syn can influence the distribution and secretion of α-syn in human neuroblastoma cells. Different α-syn variants, including α-syn:hemi-Venus and disease-causing mutants, were overexpressed and EVs were isolated from the conditioned medium. Of the secreted α-syn, 0.1–2% was associated with vesicles. The major part of EV α-syn was attached to the outer membrane of vesicles, whereas a smaller fraction was found in their lumen. For α-syn expressed with N-terminal hemi-Venus, the relative levels associated with EVs were higher than for WT α-syn. Moreover, such EV-associated α-syn:hemi-Venus species were internalized in recipient cells to a higher degree than the corresponding free-floating forms. Among the disease-causing mutants, A53T α-syn displayed an increased association with EVs. Taken together, our data suggest that α-syn species with presumably lost physiological functions or altered aggregation properties may shift the cellular processing towards vesicular secretion. Our findings thus lend further support to the tenet that EVs can mediate spreading of harmful α-syn species and thereby contribute to the pathology in α-synucleinopathies.

**Electronic supplementary material:**

The online version of this article (10.1007/s10571-018-0622-5) contains supplementary material, which is available to authorized users.

## Background

Parkinson’s disease (PD) and dementia with Lewy bodies (DLB) are the two most common disorders with Lewy body and Lewy neurite pathology. Such intracellular inclusions mainly consist of misfolded, aggregated forms of α-synuclein (α-syn) (Spillantini et al. 1997) and recent research has indicated that various soluble α-syn oligomers are the most toxic species, leading to neuroinflammation (Lee et al. 2010), oxidative stress (Unal-Cevik et al. 2011), and neurotoxicity (Diogenes et al. 2012; Winner et al. 2011).

The presence of α-syn inclusions in fetal tissue grafts 10–15 years after transplantation in PD patients suggests that cell-to-cell propagation occurs in the affected brain (Kordower et al. 2008; Li et al. 2008). In addition, accumulating evidence from cell-based studies suggests that α-syn can spread readily between cells (Domert et al. 2016; Hansen et al. 2011). It has also been demonstrated that transfer can occur not only between neurons, but also from neurons to glial cells (Reyes et al. 2014) as well as between glial cells (Rostami et al. 2017). Oligomers of α-syn seem to be particularly prone to spread (Danzer et al. 2012) and may also have a capacity to sequester monomeric protein in recipient cells, suggesting that they can be responsible for a prion-like propagation of pathology (Danzer et al. 2009). Via such mechanisms, α-syn could spread from one brain region to another, which would explain the hierarchical pattern by which pathology occurs in the PD brain (Braak et al. 2006b). Moreover, the discovery of early α-syn pathology in the nerve plexus of the gastrointestinal tract suggests that pathology may even initiate outside the central nervous system (CNS) and transfer to the brain stem via enteric neurons (Braak et al. 2006a).

It is not known by which mechanisms α-syn can spread between cells, but it has been suggested that one route may be via active secretion to the extracellular space, followed by ingestion of recipient cells (Reyes et al. 2015). Such intercellular transport could take place via conventional exocytosis or via exosomes and other extracellular vesicles (EVs) (Faure et al. 2006). Exosomes are intraluminal vesicles of endocytic origin (Valadi et al. 2007), secreted via multivesicular bodies (Subra et al. 2010), whereas other EVs are budding directly from the cell membrane. Exosomes are believed to work as vectors of cell-to-cell transmission of RNA (Valadi et al. 2007), proteins and lipids (Subra et al. 2010).

Previous studies suggest that α-syn is present in exosomes from cultured cells and that the formation of oligomers (Danzer et al. 2012) and other aggregates (Grey et al. 2015; Lee et al. 2005) can be enhanced in this environment. When inhibiting lysosomal action in α-syn overexpressing neural cell lines, exosomal secretion of α-syn increased and promoted cell-to-cell transfer of α-syn (Alvarez-Erviti et al. 2011). Moreover, cell-derived exosomes containing α-syn were found to induce cell death in neuronal cells (Emmanouilidou et al. 2010).

Alpha-synuclein can be detected both within and on the outside of exosomes and other extracellular vesicles (Danzer et al. 2012), suggesting that EV secretion is a plausible mechanism for spreading of toxic α-syn species between cells in the CNS. However, the underlying mechanisms regulating the sorting and releasing of α-syn into EVs are still poorly understood.

Different forms of α-syn may be sorted for EV release at different rates. For example, the various forms of fluorescently labeled α-syn that are often used in ex vivo experimental models for α-syn oligomerization may be differently processed by the cells compared to the physiological protein forms. It is also possible that the different α-syn mutants that cause familial forms of Lewy body disorders (reviewed in Lill 2016) may differ from the non-mutated form in this respect. Moreover, it is not clear if this regulation is dependent on the presence of other proteins, such as the PD-related molecules leucine-rich repeat kinase 2 (LRRK2) (Xiong et al. 2010), vacuolar protein sorting-associated protein 35 (VPS35) (Zavodszky et al. 2014), and ATP13A2 (Tsunemi et al. 2014).

In this study, we wanted to assess how different forms of α-syn are processed by cells for EV or non-vesicular secretion. For this purpose, we investigated cultured human neuroblastoma cells overexpressing regular wild-type α-syn as well as α-syn incorporated in a fluorescent oligomerization assay or containing any of the six disease-causing mutations (Lazaro et al. 2016, 2014). We also wanted to analyze if vesicular-associated α-syn could be internalized in recipient cells to a greater extent than secreted free-floating α-syn.

## Materials and Methods

### Cell Culture and Transient α-Synuclein Overexpression

**Fig. 1:**
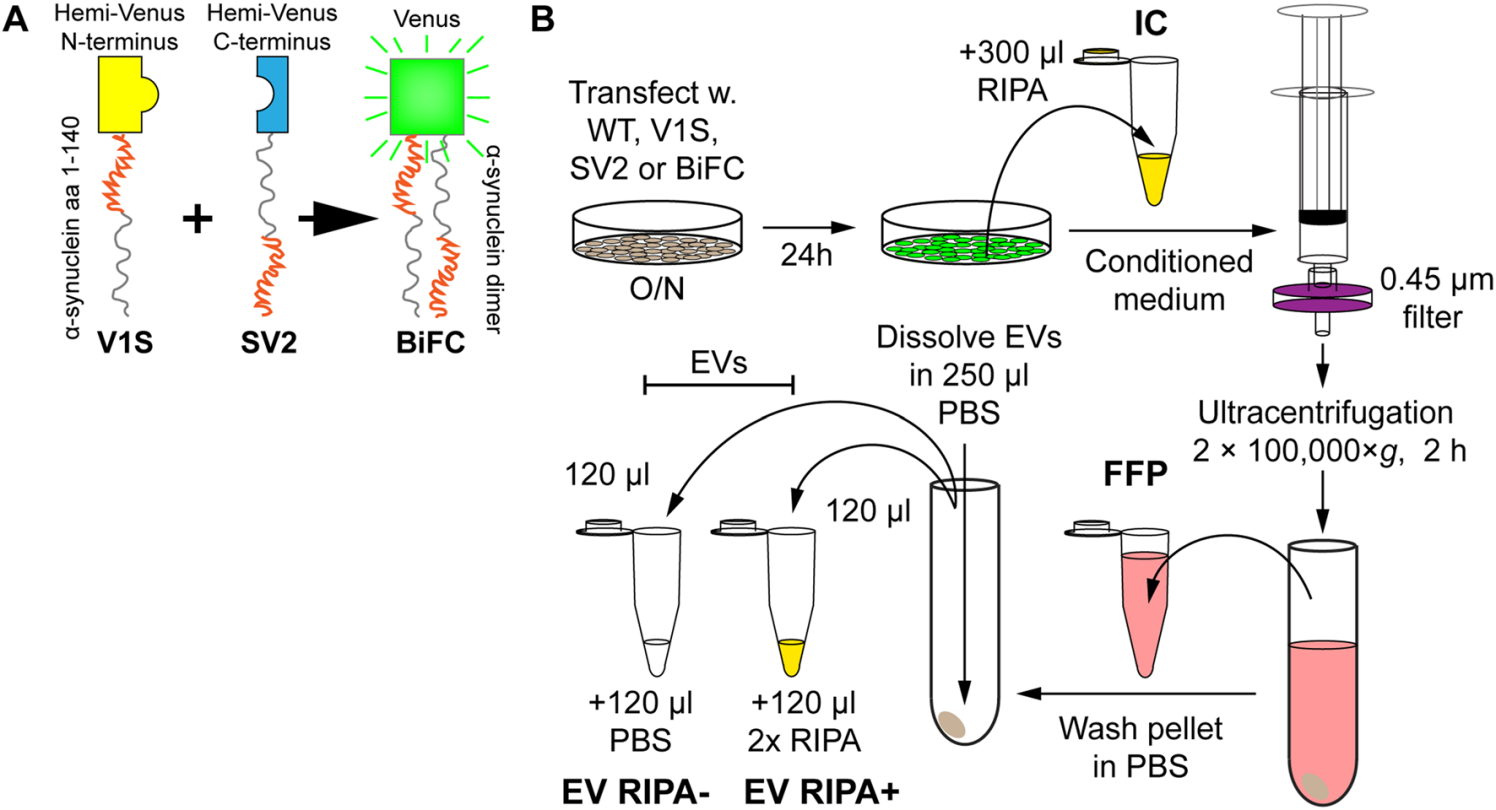
Preparation of cell-derived samples for the study of α-syn secretion. **a** In addition to human WT α-syn, the V1S (yellow) and SV2 (blue) constructs (α-syn fused with either half of Venus) were used. Also, V1S and SV2 were co-transfected in a BiFC. The N-terminal region of the α-syn portion is shown in red, whereas the C-terminal region is shown in gray. Upon α-syn dimerization of V1S and SV2, the protein aggregate fluoresces (green). **b** SH-SY5Y cells were transfected overnight. The cells were washed once in medium, followed by incubation for 24 h. The ensuing medium was collected, filtered to remove dead cells and debris, ultracentrifuged, upon which the transfected cells were washed in PBS and lysed in RIPA for the IC fraction. The medium supernatant, from the ultracentrifugation (FFP) was saved, after which the pellet was washed once followed by an exchange of tubes before the second ultracentrifugation. The ensuing pellet was reconstituted in PBS, split in two before adding either 2 × RIPA at a 1:1 ratio (EV RIPA+) or additional PBS at a 1:1 ratio (EV RIPA−), to get the two EV fractions

The human neuroblastoma cell line SH-SY5Y (94,030,304, Sigma-Aldrich, Saint Louis, MO) was used for transient α-syn expression. Cells were cultured in Opti-MEM-reduced serum medium supplemented with 5% FBS (SV30160.03, HyClone, GE Healthcare, Chicago, IL) and Penicillin/Streptomycin (15,140,122, Life Technologies, Carlsbad, CA). For transfections, cells were seeded in 60 mm Petri dishes and transiently transfected with plasmids encoding the different *α-syn* constructs. Lipofectamine 2000 (11,668,030, Life Technologies) was used for the transfections. The plasmids used were pcDNA3.1+ with the following inserts: wild-type *α-syn* (WT), hemi-peptides of Venus yellow fluorescent protein (YFP) fused to full-length wild-type *α-syn* (Venus 1–157 N-terminally fused to *α-syn* (V1S), Venus 158–238 C-terminally fused to *α-syn* (SV2) or V1S + SV2 at an equal ratio (BiFC)) (Fig. 1a), *α-syn*-2A full-length green fluorescent protein (GFP), or *α-syn* with any of the six disease-causing point mutations (A30P, E46K, H50Q, G51D, A53E, and A53T). The total amount of DNA was kept constant for both single and double transfections. After overnight (O/N) transfection, cells were washed and kept in medium with 5% FBS for 24 h. The FBS had been vesicle-depleted by ultracentrifugation at 4 °C at 120,000×*g* for 17 h, in a fixed angle rotor (Ti70, Beckman Coulter, Brea, CA).

### Tau Expression

Tau proteins were expressed with or without a GFP tag. This protein makes a relevant control since it is expressed intracellularly and forms both oligomers as well as larger aggregates (as neurofibrillary tangles) (Lasagna-Reeves et al. 2012). Plasmids encoding tau or tau fused to full-length GFP were transfected as described above.

### Sample Preparation

To remove dead cells and debris, the conditioned medium was filtered through a 0.45 µm syringe filter (2,542,903, PerkinElmer, Waltham, MA) and stored at − 20 °C. For intracellular (IC) protein analysis, cells were lysed for 30 min on ice in 1x RIPA buffer (ab156034, Abcam, Cambridge, UK) with a protease inhibitor cocktail (78,430 Thermo Fisher Scientific, Waltham, MA) and stored at − 70 °C. Prior to analysis, the lysate was thawed on ice and centrifuged at 12,000×*g*, 4 °C for 30 min. The supernatant was transferred to a new tube and stored at − 20 °C as the IC fraction. The conditioned medium was thawed and centrifuged at 100,000×*g* at 4 °C for 2 h in a fixed angle rotor. The supernatant was collected as the extracellular free-floating protein (FFP) fraction. The pellet was washed once in 500 µl PBS, which had been filtrated twice in a 0.22 µm Millex syringe filter unit (Millipore, Burlington, MA), transferred to a new centrifuge tube, and recentrifuged as above. The ensuing pellet, containing enriched EVs, was resuspended in PBS (0.1% BSA) and split in two equal volumes. These were then diluted in either PBS (0.1% BSA) or in RIPA buffer with the protease inhibitor cocktail (Thermo Fisher Scientific) to generate the two respective EV fractions (RIPA− and RIPA+) (Fig. 1b).

For the uptake experiments, the same protocol as above was used, except that no detergent was applied.

### Western Blot

After ultracentrifugation, the pellets were lysed directly in a total volume of 150 µl RIPA buffer with a protease inhibitor cocktail (Thermo Fisher Scientific). The same volumes of the original samples were added to each well. A total sample volume of 60 µl of EV and FFP fractions was run under reducing conditions on a 4–12% Bis-Tris gel for approximately 80 min at 150 V. Gels were transferred to a nitrocellulose membrane. Ponceau S was used to ensure proper transfer. Blocking was carried out at room temperature (RT) for 1 h with 5% dry milk in TBS-T before incubation with primary antibodies and after stripping with 0.4M NaOH at RT for 5 min. The membranes were incubated with primary antibodies against L1CAM (ab3200, Abcam), Alix (sc-53538, Santa Cruz Biotechnology, Dallas, TX), Flotillin-1 (610820, BD Biosciences), and CD63 (sc-15363, Santa Cruz Biotechnology). All antibodies were incubated in 5% milk in TBS-T (1:1000) O/N at 4 °C. HRP-linked secondary antibodies against rabbit (GE Healthcare) or mouse (Bio-Rad, Hercules, CA) IgG were used at a 1:20,000 dilution in 5% milk at RT for 1 h and ECL Prime (GE Healthcare) was used for development on Amersham Hyperfilm ECL (GE Healthcare).

### NanoSight Analyses

The average size and the standard deviation of the EVs in the EV and FFP fractions was determined using NanoSight (Malvern Instruments, Malvern, UK) from one example series of transfected cells.

### Transmission Electron Microscopy (TEM)

Conditioned medium was collected from SH-SY5Y cells after 24 h of incubation and fixed in 2.5% glutaraldehyde at 4 °C for 3 d. The solution was filtrated through a 0.45 µm syringe filter (Millipore) and centrifuged at 100,000×*g* at 4 °C for 2 h. The supernatant was analyzed as the FFP fraction. The pellet was resuspended in glutaraldehyde and centrifuged as above. The final pellet was resuspended in glutaraldehyde and analyzed as the EV fraction. All samples were kept at 4 °C until TEM analysis. Solutions were diluted 1:2 and stained with uranyl acetate. The samples were imaged with an H-7100 transmission electron microscope (Hitachi, Chiyoda, Japan).

### Alpha-Synuclein ELISA

High-binding half area plates (Costar, Sigma-Aldrich) were coated overnight with Syn-1 (Clone 42, BD Biosciences, San Jose, CA) as capturing antibody (50 ng/well, 610,787, BD Biosciences), diluted in PBS. Blocking with 1% bovine serum albumin was incubated on shaking for 2–4 h. Samples were added to wells and incubated at RT, shaking for 2 h, or at 4 °C O/N. The IC fractions were diluted 1000–2000 times and the FFP fractions 20–200 times. The EV fractions were not diluted prior to ELISA. Alpha-synuclein monomer standards (1.95–125 pM) were diluted in either RIPA lysis buffer or in regular ELISA incubation buffer. The polyclonal FL-140 (50 ng/well, sc-10,717, Santa Cruz Biotechnology) was used as primary detection antibody. For secondary detection, a goat anti-rabbit HRP antibody (1:5000, 31,460, Thermo Fisher Scientific) was applied followed by the K-blue aqueous substrate (TMB). Finally, 1 M H_2_SO_4_ stop solution was added and absorbance was read at 450 nm (Infinite M1000, Tecan, Männedorf, Switzerland). Three independent experiments were analyzed and samples were run in duplicates.

### Tau ELISA

The total concentrations of human tau in the samples were determined with the Tau Human ELISA kit (#KHB0041, Thermo Fisher Scientific), according to the manufacturer’s instructions. Samples were mixed with diluent buffer provided by the ELISA kit (FFP; 1:10,000, IC; 1:5000, and EV fractions; 1:2). Three independent experiments were analyzed in duplicates.

### Alpha-Synuclein Transfer Experiments

Human SH-SY5Y neuroblastoma cells were transfected with either V1S + SV2 (BiFC) or only V1S O/N and, after washing, incubated at 37 °C O/N in EV-depleted medium. The next day, the conditioned medium was collected from the donor cells and prepared as described above (see “Sample Preparation”) from four independent cultures per transfection. The samples were diluted according to the results of the α-syn ELISAs; the supernatant from the first ultracentrifugation (FFP fraction) was diluted 1:16 in EV-depleted medium and the pellet (EV fraction) was diluted 1:2 in EV-depleted medium. Non-transfected recipient cells were then incubated with 500 ul medium with either FFP or EV fractions at 37 °C O/N. At 24 h post donation, recipient cells were washed twice in PBS to wash away non-engulfed protein and fixed in 4% paraformaldehyde, blocked, followed by permeabilization in 5% normal goat serum in 0.1% Triton-X in PBS. The cells were incubated with primary anti-GFP polyclonal antibodies (1:400, ab290, Abcam) at RT for 2 h, followed by incubation with fluorescent secondary antibodies (Alexa Fluor 488-linked goat anti-rabbit; 1:1000, A-11,008, Thermo Fisher Scientific) at RT for 1 h. Slides were mounted in HardSet Vectashield mounting medium containing DAPI (H-1500, Vector Laboratories), dried, and imaged with a Zen microscope (Carl Zeiss, Oberkochen, Germany) with a × 40 objective. A total of 22–25 images per well of the independent treatments were taken using the same settings and analyzed using a macro in ImageJ with preset, consistent thresholds for all images. The resulting area of the GFP staining was normalized against the number of cells, which in turn was normalized against the mean area per cell from non-recipient, control cells (blank, *n* = 4).

### Analyses and Statistics

For the α-syn ELISA, a mean value was calculated from the duplicate wells. The two α-syn concentration standards made in different buffers were applied to calculate α-syn levels in the four different fractions. For IC, FFP, and RIPA− EV fractions, concentrations were estimated from the standard curve made from dilutions in regular ELISA incubation buffer. The RIPA+ EV fractions were calculated from the standard curve made from dilutions in 1xRIPA lysis buffer. Total concentrations are presented as either pg/ml or pM. The ELISA data displayed a normal distribution and one-way ANOVA with Dunnett’s multiple comparison test was carried out with the WT sample as control (GraphPad Prism). Levels of significance were set to *p < 0.05, **p < 0.01, ***p < 0.001. Pairwise t-test comparisons between absolute values of RIPA+ and RIPA− were also made where the levels of significance were set to the same criteria.

For the uptake experiments, the normalized values were analyzed in GraphPad Prism by one-way ANOVA with Tukey’s post hoc test. Significance levels were set to *p < 0.05, **p < 0.01, and ***p < 0.001.

## Results

### Isolation of Extracellular Vesicles from Neural Cell Lines

**Fig. 2:**
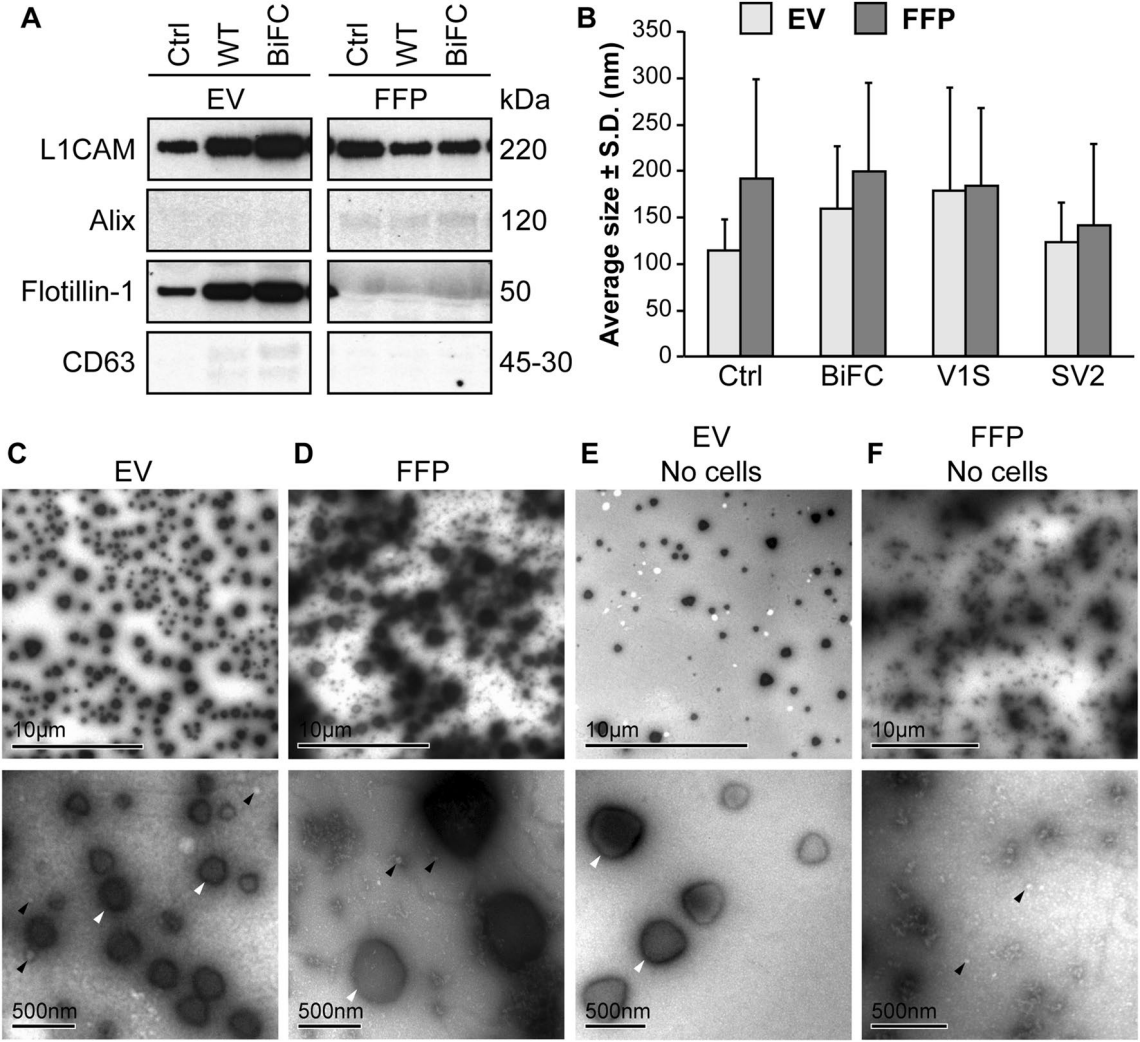
The EV fractions are enriched in exosomal markers. **a** Western blots of the lysed EVs and the corresponding FFP fractions were analyzed for presence of the markers L1CAM, Alix, Flotillin-1, and CD63. Flotillin-1 was the only marker that was highly enriched in all the EV fractions compared to the FFP fractions, but also CD63 was slightly enriched in EVs, indicating that different markers may be differentially present in different subclasses of EVs. The membrane images have been cut to exclude samples outside the scope of this project. Unbiased contrast changes have been made to the images, but the unchanged, whole membrane is shown in Supplementary Fig. 1. **b** NanoSight analysis of EVs and FFP fractions showed similarly sized particles in samples from non-transfected (Ctrl) and tagged α-syn transfected cells with an average diameter of approximately 150 nm. The FFP fraction also contained particles of a similar size, albeit to a much lesser degree than the EV fraction. **c** TEM analysis of the EV-enriched fraction displayed EVs with diameters between 50 and 500 nm. Both large high density (white arrowheads) and small less dense (black arrowheads) particles were highly enriched. **d** TEM analysis confirmed the NanoSight results, where small EVs (white arrowheads) and large, dense particles (black arrowhead) were found in the FFP fraction. **e** Ultracentrifuged growth medium containing EV-depleted FBS also displayed the large particles in the EV fraction (white arrowheads), albeit to a much lesser degree than the EV fraction. **f** A few small EVs (black arrowheads), but no large vesicles, were found in the FFP after ultracentrifugation of the FBS-depleted growth medium

In order to characterize the EV content in the SH-SY5Y cell-derived extracellular fractions (EV; extracellular vesicles and FFP; free-floating protein), we investigated various markers by Western blot. The EV fractions from controls and *α-syn* transfected cells displayed clear bands for L1CAM and Flotillin-1 (Fig. 2a). The FFP fractions, however, did not seem to contain any Flotillin-1. CD63 was only modestly represented in the EV fractions and not at all in the FFP fractions. In contrast to the other markers, Alix was not found in the EV fractions and only weakly in the FFP fractions (Fig. 2a). The size distribution of the isolated vesicles was determined by NanoSight and revealed a size range of about 100–300 nm (Fig. 2b). Furthermore, analyses of EV fractions with TEM displayed enrichment of vesicles in the same size spectrum as detected by NanoSight (Fig. 2c). The enrichment of Flotillin-1 along with an apparent size distribution of 100–300 nm suggests that the enriched ultracentrifugation pellet contained a mixture of exosomes and other EVs. We have therefore defined the vesicles in the enriched fractions with the general term *extracellular vesicles*. The FFP fractions contained debris and only a small amount of vesicles (Fig. 2d). Serum-depleted growth medium that had not been in contact with any cells was ultracentrifuged to analyze the background levels of EVs and FFPs. The EV fraction from the cell-free medium also displayed vesicles, albeit to a much lesser extent than that which had been collected from cells (Fig. 2e). The corresponding cell-free FFP fraction contained particles and very few vesicles (Fig. 2f).

### Tagged α-Syn is Directed to EV-Mediated Secretion

**Fig. 3:**
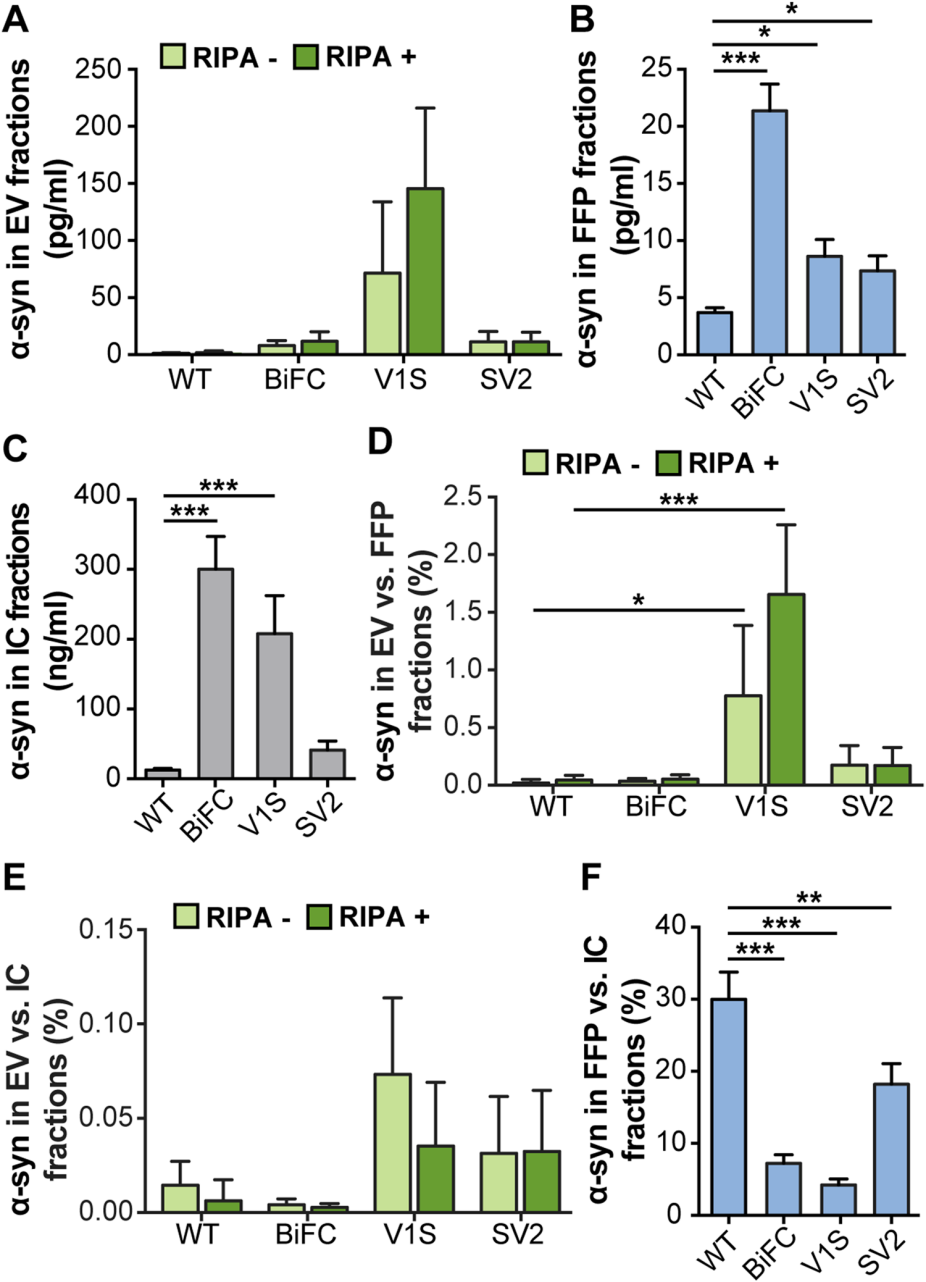
N-terminally tagged α-syn is enriched in the EV fraction. **a** Absolute levels of α-syn in EV (light green: RIPA−, dark green: RIPA+), **b** FFP fractions, and **c** IC fractions, as measured by ELISA. **d** Percentage of α-syn in EV fractions compared to FFP fractions. **e** Percentage of α-syn in EV compared to IC fractions. **f** Percentage of α-syn in FFP compared to IC fractions. The distribution of V1S differed from the other α-syn species. Lysis of the vesicles did not affect the levels of α-syn, indicating that the protein was located mainly on the outside of the EVs. Bars represent mean ± SD of three independent experiments. Statistical significance was calculated by one-way ANOVA with Dunnett’s post hoc test, with levels compared to those of WT α-syn (*p < 0.05, **p < 0.01, ***p < 0.001)

To assess the distribution of α-syn after expression of the different tagged constructs, fractions separated with ultracentrifugation and intracellular (IC) fractions were analyzed by a total α-syn sandwich ELISA. The ELISA signals in EV fractions varied between different transfections. In all samples, the RIPA+ signal was equal to, or only slightly higher, than the corresponding RIPA− signal, indicating that α-syn was mostly present on the outside of the membrane but to some extent also resided inside the EVs (Fig. 3a). Expression of V1S, SV2, as well as co-expression of V1S and SV2 led to higher signals than WT α-syn in the FFP fraction (Fig. 3b). Expression of V1S as well as co-expression of V1S and SV2 led to higher signals than WT α-syn in the IC fraction (Fig. 3c).

In order to normalize for transfection differences between the constructs, the relative levels of α-syn in the different fractions were calculated as ratios of the EV fraction over FFP (Fig. 3d) or IC fractions (Fig. 3e). The ratio between EV and FFP fractions revealed a significant increase with the V1S construct (Fig. 3d), compared to WT-transfected cells. Thus, the N-terminal tag on α-syn seemed to cause a marked redirection in the cellular processing of α-syn. When EVs were normalized against IC fractions, V1S also gave the highest ratio of α-syn in EVs (Fig. 3e), although the difference between the species was smaller. In contrast to the tagged α-syn, the ratios of FFP versus IC fractions of the WT samples were significantly higher than for the other samples, indicating that the WT protein was secreted in the FFP fraction rather than via EVs (Fig. 3f).

### Tau Secretion in EVs is Not Affected by GFP Fluorescent Tag

Based on our observation of increased levels of V1S in EVs versus FFPs as compared to WT α-syn, we wanted to explore whether a similar shift could be observed also for fluorescently labeled tau, another protein involved in neurodegeneration. Non-tagged tau and tau:GFP were expressed in SH-SY5Y cells. The EV-enriched and FFP fractions from the overexpressing cells were analyzed by ELISA.

**Fig. 4:**
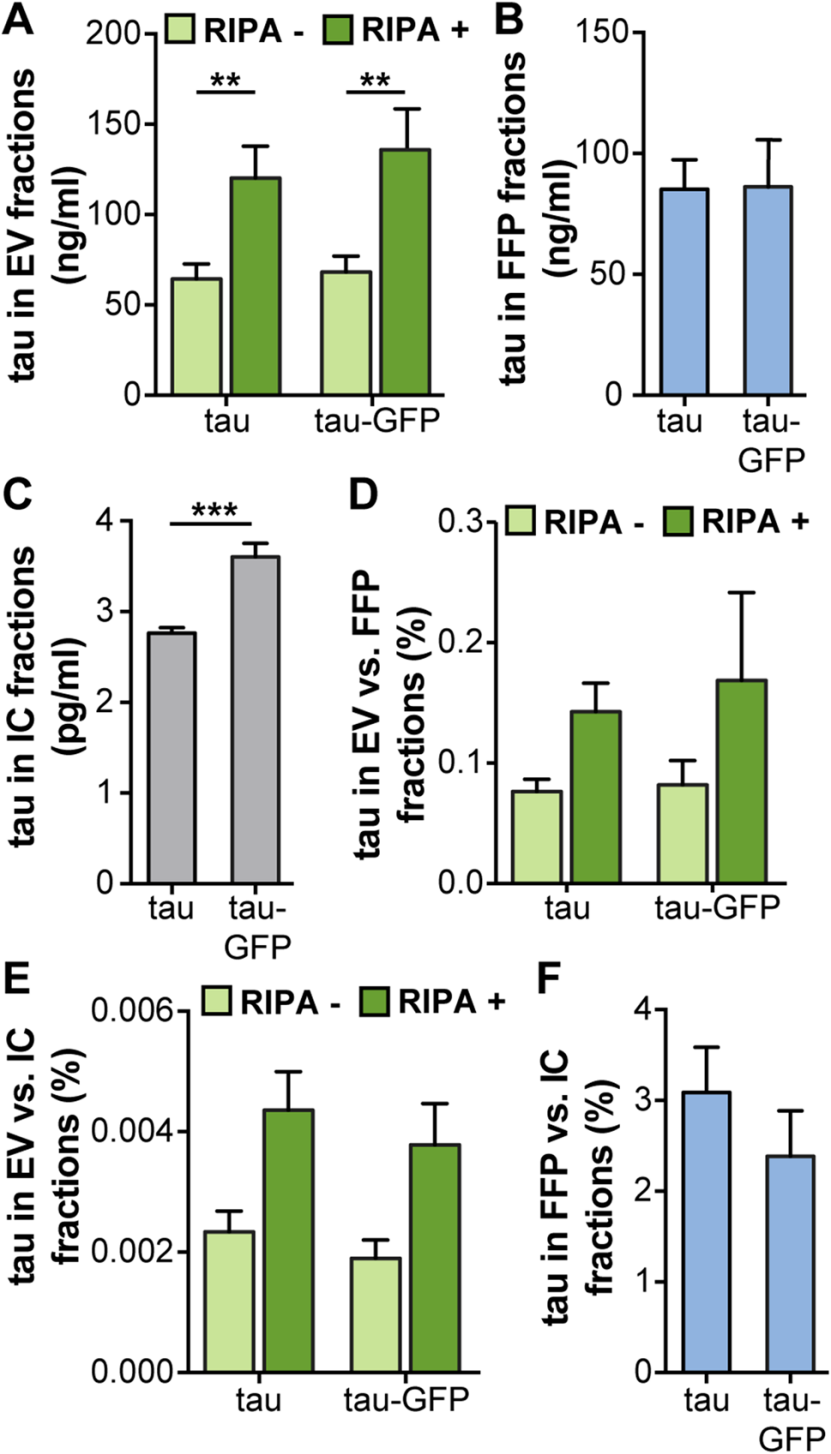
No difference in tagged versus untagged tau in EV fractions. In order to investigate whether also tau is differentially processed via EVs when modified, cells were transfected with either tau or tau:GFP. **a** Absolute levels of tau in EVs, **b** FFP, and **c** IC fractions, as measured by ELISA. **d** Percentage of tau in EVs compared to FFP fractions. **e** Percentage of tau in EVs compared to IC fractions. **f** Percentage of tau in FFP compared to IC fractions. In contrast to α-syn, the relative distribution of tau did not change when the GFP tag was present. Lysis of the vesicles significantly increased the levels of tau, indicating a large portion of the protein inside the EVs. Bars represent mean ± SD, *n* = 3. A *t* test was used to determine the significance between the two constructs (*p < 0.05, **p < 0.01, ***p < 0.001)

Lysis of the EVs with RIPA buffer resulted in a significant increase in signals for both tau constructs (Fig. 4a). Signals were equal in the FFP fractions (Fig. 4b), whereas the tau:GFP signal was significantly higher in the IC fractions (Fig. 4c). Normalization of EV fractions against FFP (Fig. 4d) or IC fractions (Fig. 4e) showed no significant difference of tau with or without GFP tag (Fig. 4d, e). In addition, comparisons between the amount of tau in FFP and IC showed similar ratios (Fig. 4f), indicating that both tau constructs were processed similarly in terms of secretion via EVs or as FFPs in the cell culture medium.

### EV-Associated N-Tagged α-Syn has an Increased Cellular Uptake

**Fig. 5:**
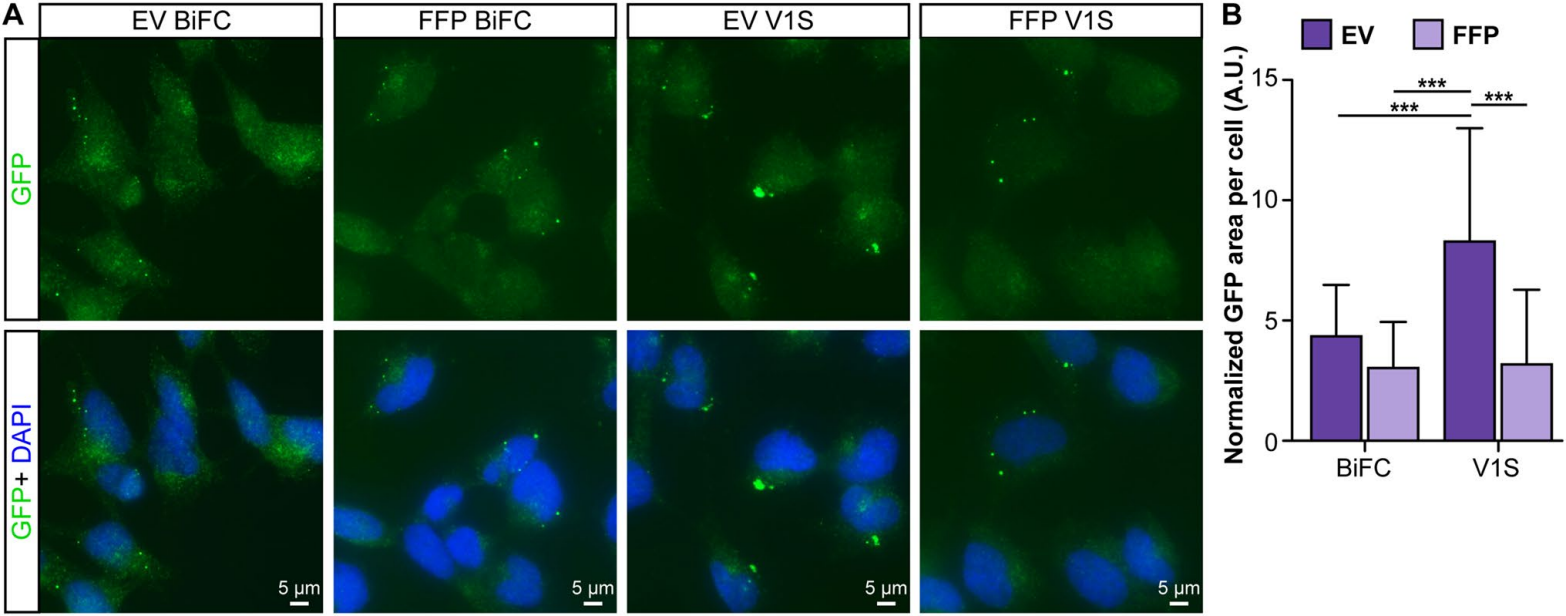
EV-associated, V1S-tagged α-syn is readily taken up by recipient cells. **a** Example images from cells receiving EV or FFP fractions from cells expressing V1S + SV2 (BiFC) or V1S only. Conditioned medium was added to non-transfected, SHSY5Y recipient cells for 24 h. The cells were washed twice in PBS and, following fixation, immunocytochemistry against the hemi-Venus tag was performed to allow visualization and display both constructs independently of dimerization (green). **b** The EV-associated V1S-tagged α-syn was taken up or retained to a significantly greater extent than the other fractions. Dark purple bars represent the EV fractions and the light purple bars represent the FFP fractions (mean ± SD, n = 4). One-way ANOVA with Tukey’s post hoc test was used to determine the differences between the groups (*p < 0.05, **p < 0.01, ***p < 0.001)

To investigate cell-to-cell transfer of EV-associated α-syn, donor cells were double transfected with the *α-syn*:hemi-Venus plasmids V1S + SV2 or with V1S only. Conditioned medium was collected and fractions were isolated from four independent transfections per construct. Non-transfected cells received either EV or FFP fractions for 24 h. To discriminate between endogenous α-syn and externally applied α-syn and to enhance the signal from the internalized protein, the cells were stained with a polyclonal GFP antibody that binds to both V1S and SV2. Small punctae could be visualized in the recipient cells at 24 h post treatment (Fig. 5a) and the area difference per number of cells was analyzed after being normalized against background staining (data not shown). The staining analysis revealed a markedly increased uptake or prolonged retention of EV-associated V1S, as compared to all other fractions (Fig. 5b, *p* < 0.001).

### A53T α-Synuclein is Directed Towards EV Secretion

**Fig. 6:**
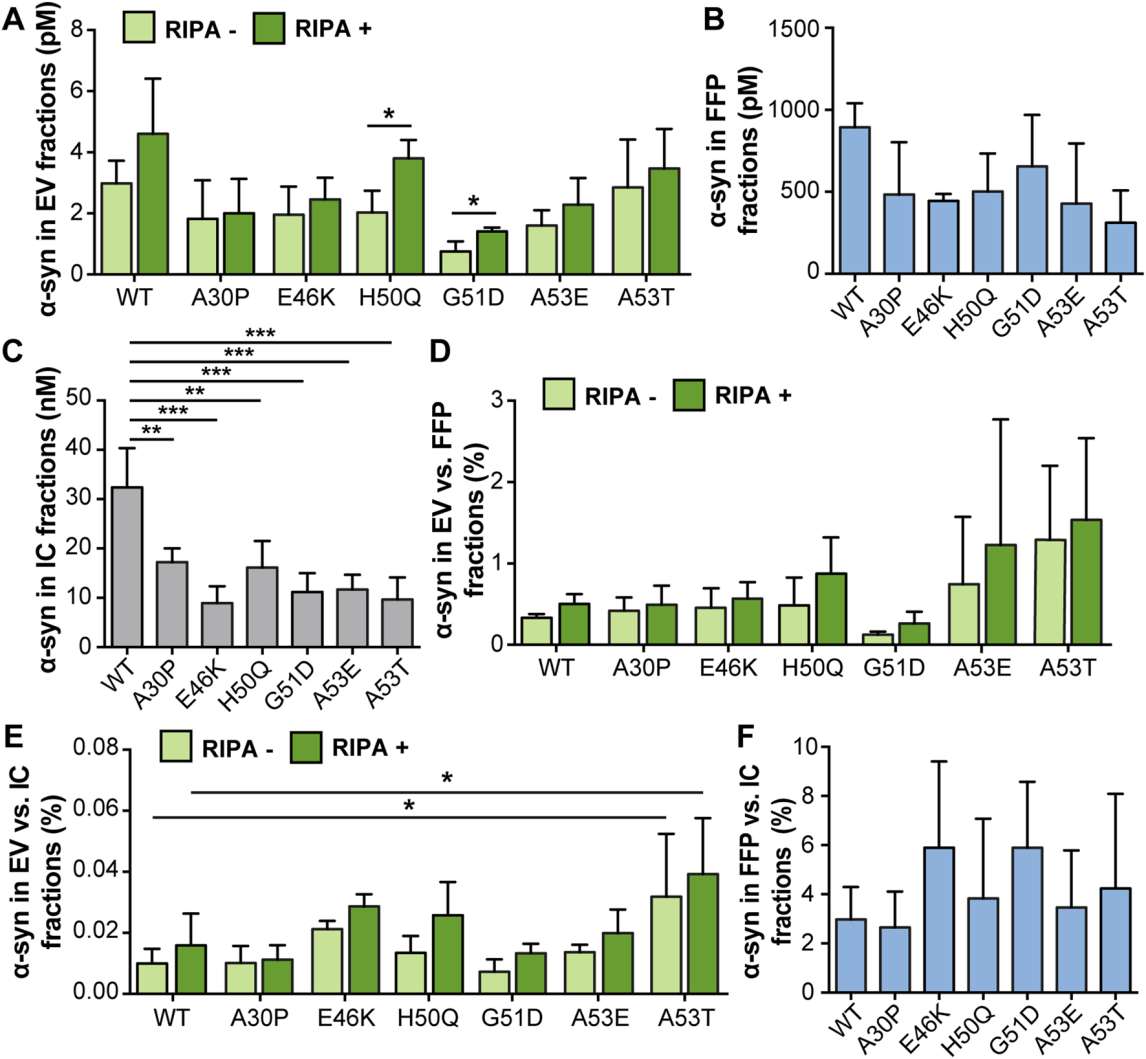
A53T α-syn was enriched in the EV fraction. **a** Absolute levels of α-syn in EV (light green: RIPA–, dark green: RIPA+), **b** FFP and **c** IC fractions, as measured by ELISA. **d** Percentage of α-syn in EV compared to FFP fractions. **e** Percentage of α-syn in EV compared to IC fractions. **f** Percentage of α-syn in FFP compared to IC fractions. The distribution of A53T differed from WT α-syn and the other mutants and was higher in both RIPA+ and RIPA− fractions when normalized against IC values. Bars represent mean ± SD, *n* = 3. Differences between the respective RIPA− and RIPA+ fractions from WT α-syn were calculated by one-way ANOVA with Dunnett’s post hoc test (*p < 0.05, **p < 0.01, ***p < 0.001)

Cells were transfected with plasmids encoding the different familial α-syn mutants as well as WT α-syn. Measurements by ELISA indicated that the α-syn levels in EV fractions from the mutants were comparable to those for WT α-syn expression where most of the α-syn was present on the outside of the EVs (Fig. 6a). The total α-syn levels in the EV fractions were not significantly affected for any of the mutations, as compared to WT α-syn. However, the H50Q and G51D mutants displayed significantly increased α-syn signals in the lysed EVs (RIPA+), when compared to their corresponding native (RIPA−) EV samples, which could be a result of differences in binding affinity to lipid membranes (Stefanovic et al. 2015). Levels in the FFP fractions were comparable across all cells expressing either mutant or WT protein (Fig. 6b). The WT α-syn transfection led to significantly higher IC levels of α-syn compared to transfection of mutation constructs (Fig. 6c). The ratios of EV values were in the range of 0.1–2% compared to FFP fractions and 0.01–0.05% compared to IC fractions (Fig. 6d, e, respectively). For A53T α-syn, there was a trend for an increased ratio of EV to FFP α-syn levels (Fig. 6d) and a statistically significant increase in the ratio of EV to IC α-syn levels compared to WT α-syn (Fig. 6e), both for the RIPA+ and RIPA− EV fractions. The ratio of FFP to IC α-syn was in the same range for all constructs with and without *α-syn* mutations (Fig. 6f).

## Discussion

Our study lends support to the notion that exosomes and other EVs are involved in the spreading and/or seeding of α-syn, similarly to what others have found (Alvarez-Erviti et al. 2011; Danzer et al. 2012). The vesicles that were collected from the cell medium in the present study displayed various sizes and were strongly displaying Flotillin-1, which suggests that they represent a mixture of exosomes and other EVs. Therefore, the vesicular secretion of α-syn could be attributed to either one or several types of vesicles. The vesicles found in the EV and FFP fractions were of a similar size range (Fig. 2b–d), implying that the used protocol leads to an enrichment of EVs rather than a separation of large and small vesicles.

This study indicates that distinct presumably dysfunctional forms of α-syn may alter the cellular dynamics. Most notably we found that V1S, one of the two α-syn:hemi-Venus forms used in the BiFC assay for α-syn oligomerization, was particularly prone to be directed for EV secretion, whereas the other form, SV2, did not seem to be secreted differently than regular α-syn. The underlying reason for the observed differences between the two α-syn:hemi-Venus species with respect to EV secretion is unclear, but could possibly be due to alterations in membrane-binding properties. It has previously been shown that fluorescent protein tags on the N-terminus of α-syn can alter the intracellular dynamics (Goncalves and Outeiro 2013) and induce vesicular secretion (Jang et al. 2010) of α-syn. In the latter study, it could also be shown that, depending on the nature of the tag, α-syn is more or less sensitive to Proteinase K (Jang et al. 2010). Those results and the data from the current study thus indicate that N-terminal protein tags on α-syn lead to altered membrane-binding properties and may form particularly pathogenic and stable forms of aggregated α-syn that could increase cell-to-cell spreading. Moreover, N-terminal glycation of α-syn has also been shown to reduce its membrane binding, leading to altered cellular α-syn processing, and formation of toxic oligomers (Vicente Miranda et al. 2017). Altogether, these findings suggest that disruption of the membrane binding of the N-terminal portion of α-syn per se, rather than the presence of a fusion protein or any other molecule, can augment its propensity for EV secretion.

In our study, differences in expression levels were normalized by analyzing ratios between the different fractions from one transfection. This normalization is crucial, allowing comparison of relative levels in EV, FFP, and IC fractions between the different α-syn species. Even though the different α-syn variants were expressed in the same type of plasmid, under the same promoter, and the amount of DNA used was kept constant, total α-syn levels varied substantially across the different species. We realize that the differences in total α-syn levels to some extent may affect the dynamics of cell distribution and processing of α-syn. On the other hand, these variations may be the biological effects of the respective α-syn variants and could be caused by differential cellular accumulation or degradation as a result of variations in the oligomerization/aggregation rates. In turn, these factors could also have a direct impact on the extent of vesicular secretion of α-syn.

We could here corroborate previous findings that only a minor fraction (0.1−2%) of secreted α-syn are associated to EVs, whereas the majority of the protein can be found free in the extracellular space (Danzer et al. 2012; Shi et al. 2014). Even though the EV-associated fraction of extracellular α-syn is small, such vesicles are considered to be biologically active (van Niel et al. 2006) and molecules in this environment could be more efficiently delivered to other cells (Subra et al. 2010). In concurrence with Danzer et al. (2012), we found that certain forms of EV-associated α-syn are more prone to internalization or retention by a second generation of cells, as compared to free-floating, extracellular protein. This observation supports the notion that intercellular transfer of pathogenic forms of α-syn could be caused by EV-associated α-syn. Interestingly, vesicles have been shown to increase oligomerization of α-syn (Grey et al. 2015; Lee et al. 2005) and oligomers are known to negatively impact cellular health to a greater extent than monomers or large aggregates (reviewed in Ingelsson 2016). Although we cannot say whether the observed punctae in the recipient cells (Fig. 5a) are derived from tagged α-syn oligomers in vesicles or bona fide aggregates, they could possibly be represented by EV-associated α-syn:hemi-Venus species that are being endocytosed and processed in unknown cellular trafficking pathways (Delenclos et al. 2017).

As we wanted to explore whether the directed exocytosis of V1S merely was a consequence of the cells removing non-physiological protein forms, we also analyzed how another protein was processed with and without a similar tag. We selected tau as it, similarly to α-syn, is a protein involved in neurodegeneration that also forms intracellular oligomers and fibrils (Lasagna-Reeves et al. 2012) and that, like α-syn, has been proven to spread between cells (de Calignon et al. 2012; Takeda et al. 2015). Similar to α-syn, tau was found to be present both on the outside and the inside of the vesicles, although both tau and tau:GFP were located on the inside of the vesicles to a greater extent than α-syn. However, when comparing total as well as relative levels of tau with and without a GFP tag, we could not find any significant differences in the distribution to EV and FFP fractions between these two tau variants. These data suggest a selective mechanism for the differential distribution of V1S fusion proteins towards EVs, rather than a general phenomenon for non-physiological hybrid proteins.

Two of the α-syn mutants, H50Q and G51D, were found to end up significantly more on the inside than on the outside of the EVs. It has previously been shown that H50Q alters the interaction of α-syn with cellular membranes and increases its aggregation in the presence of Cu^2+^ (Villar-Pique et al. 2016), whereas G51D oligomers were found to have a much lower permeabilization potential compared to WT α-syn (Stefanovic et al. 2015). Such features may, at least partly, explain the differential EV localization of these two α-syn species that was seen in our study.

A53T α-syn was the only assessed mutant that led to an increase in the EV-to-IC ratio. Interestingly, the total values for A53T in the FFP and IC fractions appeared slightly lower than for other mutants. As A53T α-syn has an increased fibrillization propensity (Giasson et al. 2002; Greenbaum et al. 2005), it could for this reason be differentially processed by the cells. However, with the protocol used, it cannot be excluded that small extracellular α-syn aggregates to some extent also are enriched in the EV pellet. On the other hand, the increased α-syn ELISA signal after addition of RIPA buffer indicates that α-syn in the EV pellet is associated to lipid membrane structures. It is tempting to speculate that the increased EV secretion of A53T α-syn may be driven by an increased formation of fibrillar seeds in the overexpressing cells. Consequently, it could be hypothesized that this particular PD mutation has a higher EV contribution in the propagation of pathology. Even though the changes were relatively small, they could be detected already upon 48 h of incubation and it is plausible that this mutant may have a more pronounced long-term impact on α-syn propagation in the affected brain.

In conclusion, our study suggests that α-syn species with presumably altered physiological properties may be more prone for secretion via extracellular vesicles and thus increase cell-to-cell spreading of pathogenic proteins. Such mechanisms may contribute to the propagation of pathology in brains from patients with α-synucleinopathies.

## Electronic supplementary material

Below is the link to the electronic supplementary material.

**Supplementary Figure 1. Whole gels of the Western blot** files included in Figure 2. A) L1CAM showed a clear band at the expected 220 kDa size mark **B)** Alix only showed slight bands at the expected 120 kDa size mark. **C)** Flotillin-1 had a high presence in the EV samples at the expected 50 kDa size mark. **D)** CD63 displayed several bands around the 30-45 kDa size marks, most likely representing post-translational modifications of the protein. Note that the Flotillin-1 and CD63 blots were processed in parallel, but that the lengths of exposure differed between **C** and **D**. The rectangles marked “Not in manuscript” represent samples outside the scope of this study (TIF 3289 KB)

Supplementary material 2 (XLSX 256 KB)

## Data Availability

All data generated or analyzed during this study are included in this published article and its Supplementary Information files.
