# Supplementary figures and images for: Secretion and Uptake of α-Synuclein Via Extracellular Vesicles in Cultured Cells
Source: Cell Mol Neurobiol. 2018 Oct 4;38(8):1539–50. doi: 10.1007/s10571-018-0622-5 (PMC6223723; doi:10.1007/s10571-018-0622-5)

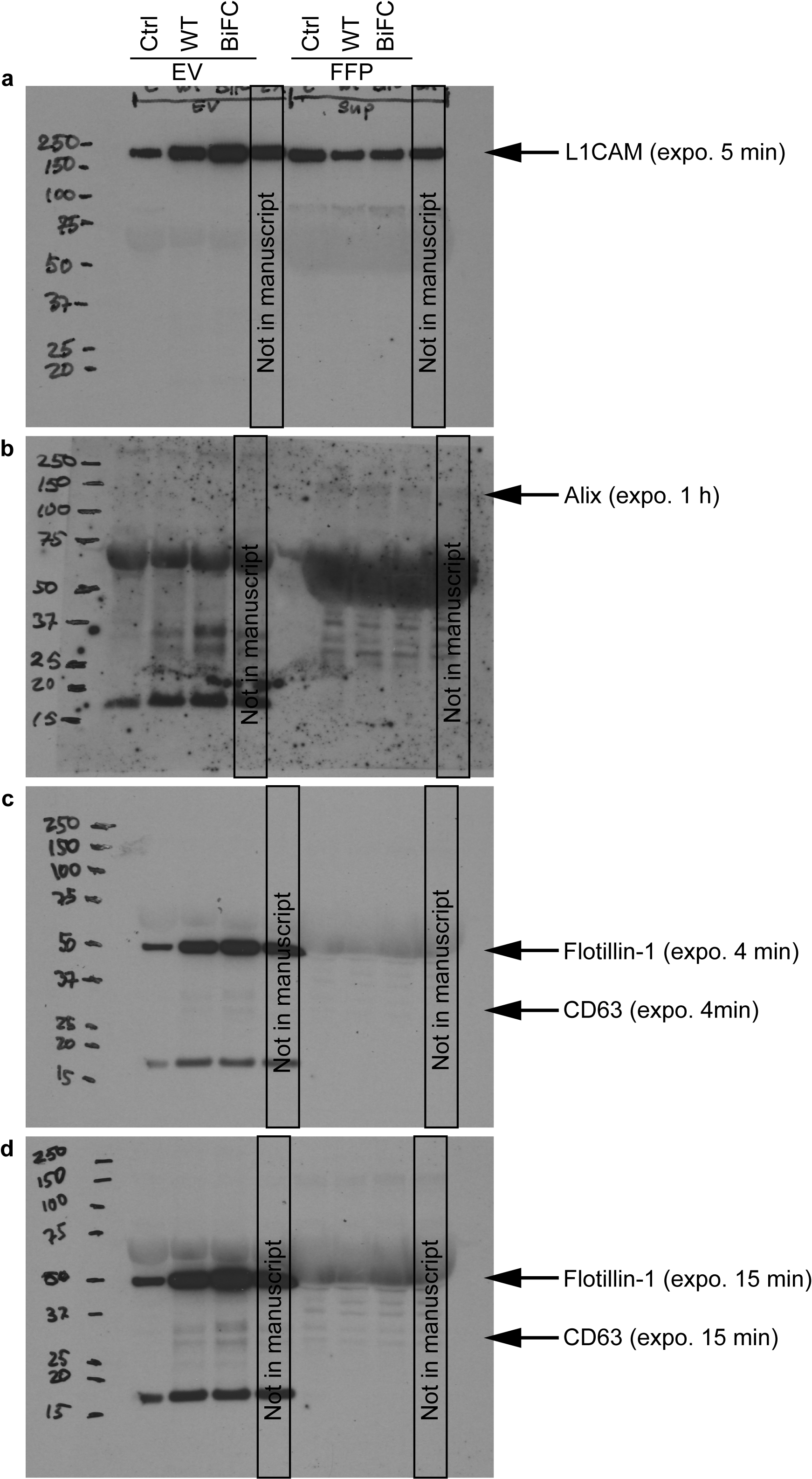

Supplement: Supplementary file 1 — Supplementary Figure 1. Whole gels of the Western blot files included in Figure 2. A) L1CAM showed a clear band at the expected 220 kDa size mark B) Alix only showed slight bands at the expected 120 kDa size mark. C) Flotillin-1 had a high presence in the EV samples at the expected 50 kDa size mark. D) CD63 displayed several bands around the 30-45 kDa size marks, most likely representing post-translational modifications of the protein. Note that the Flotillin-1 and CD63 blots were processed in parallel, but that the lengths of exposure differed between C and D. The rectangles marked “Not in manuscript” represent samples outside the scope of this study (TIF 3289 KB) [file 10571_2018_622_MOESM1_ESM.tif]
